# Life Insurance and Irregular Practitioners

**Published:** 1849-04

**Authors:** 


*Life Insurance and Irregular Practitioners.*—We find in the Boston Med. and Surg. Journal, the following extract from a private letter by the Secretary of the *American Mutual Life Insurance Company*, to the Editor. The position taken by the company is certainly reasonable and proper. Strictly speaking, as it is but justice to the profession, and a measure of sound policy on the part of the company, it can hardly be considered in the light of a compliment; yet, such is the want of decent respect for legitimate medicine by a portion of the discriminating public now-a-days, that we cordially concur in the remark of our respected contemporary, that "the brotherhood should, if they do not take out policies for themselves, use their influence in behalf of corporations that set their faces decidedly and irrevocably against all orders of quacks and quackery." There is another reason why we should pursue this course—viz: Corporations adopting similar regulations are, *ceteris paribus*, much safer and more reliable.

"It is always one of our rules to reject an insurance, if we know the family physician to be a quack, or belonging to that class of practitioners whose system of practice is to give that which would neither inconvenience a well man, nor benefit a sick one. Life Insurance Companies should look at the doctor risk, with the same care that they do a sea, or climate risk. We intend to insure none but those who have a home, friends, and a good doctor. Wanderers, the homeless, friendless, and heedless, we carefully avoid, and have rejected many applications, where the subject itself was *good*, for the reason that we were unwilling to encounter the risk resulting from personal carelessness, and the chance of being houseless, friendless, and doctorless in case of sickness. We do not allow an insured person to trifle with his life (or if he does, not to trifle with our funds,) by going into the Southern States during the sickly months, nor by wandering in unsettled portions of the United States; but all this risk is, in our opinion, not so great as that incurred by insuring a man, who, when he becomes sick, allows a certain class of doctors to trifle with his life. As we seek business only from the old and healthy States, and profess to be, and being truly, a 'perfectly Mutual Company,' it requires much care and some sagacity to preserve untarnished the standard we have adopted."

